# Supplementary material for: Maximizing protein production by keeping cells at optimal secretory stress levels using real-time control approaches
Source: Nat Commun. 2023 May 25;14:3028. doi: 10.1038/s41467-023-38807-9 (PMC10212943; doi:10.1038/s41467-023-38807-9)
Supplement: Supplementary file 1 — Supplementary Information [file 41467_2023_38807_MOESM1_ESM.pdf]

Maximizing protein production by keeping cells at optimal secretory stress levels using real-time control approaches

-Supplementary Material-

Sebastián Sosa-Carrillo[1], Henri Galez[1], Sara Napolitano[1], François Bertaux[1,2*], Gregory Batt[1*]

1. Institut Pasteur, Inria, Université Paris Cité, 75015 Paris, France
2. Lesaffre International, 101 rue de Menin, Marcq-en-Baroeul, France

* These authors contributed to this work equally.
Correspondence: gregory.batt@inria.fr

Contents

Supplementary note 1. Heterologous proteins and yeast strains 2

Supplementary note 2. Use of an accessory strain 3

Supplementary note 3. Analysis of iPOI location in the cells 5

Supplementary note 4. Quantification of secreted protein levels using magnetic immune beads 6

Supplementary note 5. Complete time-course data for iPOI and UPR levels and analysis of the accumulator population 10

Supplementary note 6. Analysis of cellular growth rates 12

Supplementary note 7. Characterization of *HAC1* knockout strains 15

Supplementary note 8. ReacSight scripts for real-time control experiments 17

Supplementary references 18

Supplementary note 1. Heterologous proteins and yeast strains

Here we provide the list of proteins we studied and the strains we used in this work. All strains are derived from the *S. cerevisiae* BY4741 strain (*MATa his3Δ1 leu2Δ0 met15Δ0 ura3Δ0*).

Table S1.1 Heterologous proteins of interest and some of their features

Protein under study	POI name	PTM*	Native organism	Size (aa)**	*GenBank* ID
Non-secreted mNeonGreen[1]	Non-sec mNeon	N/A	N/A	332	BBB44438
Secreted mNeonGreen[1]	mNeon	N/A	N/A	354	BBB44438
Endo-1,4-beta-xylanase C[2]	XylC	1x S-S	*Aspergillus niger*	662	EU848304
Endo-1,4-beta-xylanase 2[3]	Xyl2	2x N-glyc	*Trichoderma reesei*	545	P36217.2
Paraoxonase 1[4]	hPON1	1x S-S + 3x N-glyc	*Homo sapiens*	709	NM_000446.5
Single chain variable fragment 4M5.3[5]	scFv	2x S-S	N/A	609	1X9Q_A
α-amylase[6]	amy	4x S-S + 2x N-glyc	*Aspergillus oryzae*	832	CAA31220

* **PTM**: posttranslational modifications; **S-S**: disulfide bond; **N-glyc**: N-glycosylation. ** Includes, if present, the secretion tag, the protein of interest, the fluorescent reporter, and the three FLAG tag copies.

Table S1.2 Description of the yeast strains used in this study.

Name	ID	Parental strain	URA3 locus (URA3 selection)	LEU2 locus (LEU2 selection)	HO locus (HIS3 selection)	Auxotrophies
Parental	yIB32	BY4741	pTDH3 NLS-VP16-EL222 tSSA1 (pIB0120)			Leucine, Histidine, Methionine
UPR sensor	yIB90	yIB32	pTDH3 NLS-VP16-EL222 tSSA1 (pIB0120)	pUPR mScarlet-I tENO1 (pIB0115)		Histidine, Methionine
Non-sec mNeon	yIB330	yIB90	pTDH3 NLS-VP16-EL222 tSSA1 (pIB0120)	pUPR mScarlet-I tENO1 (pIB0115)	pLight mNeonGreen-3xFLAG tTDH1 (pIB0682)	Methionine
mNeon	yIB169	yIB90	pTDH3 NLS-VP16-EL222 tSSA1 (pIB0120)	pUPR mScarlet-I tENO1 (pIB0115)	pLight alpha-prepro-mNeonGreen-3xFLAG tTDH1 (pIB0399)	Methionine
XylC	yIB314	yIB90	pTDH3 NLS-VP16-EL222 tSSA1 (pIB0120)	pUPR mScarlet-I tENO1 (pIB0115)	pLight alpha-prepro-xylanase_C-mNeonGreen-3xFLAG tTDH1 (pIB0678)	Methionine
Xyl2	yIB315	yIB90	pTDH3 NLS-VP16-EL222 tSSA1 (pIB0120)	pUPR mScarlet-I tENO1 (pIB0115)	pLight alpha-prepro-xylanase_2-mNeonGreen-3xFLAG tTDH1 (pIB0679)	Methionine
hPON1	yIB170	yIB90	pTDH3 NLS-VP16-EL222 tSSA1 (pIB0120)	pUPR mScarlet-I tENO1 (pIB0115)	pLight alpha-prepro-hPON1-mNeonGreen-3xFLAG tTDH1 (pIB0400)	Methionine
scFv	yIB171	yIB90	pTDH3 NLS-VP16-EL222 tSSA1 (pIB0120)	pUPR mScarlet-I tENO1 (pIB0115)	pLight alpha-prepro-scFv-mNeonGreen-3xFLAG tTDH1 (pIB0401)	Methionine
Amy	yIB313	yIB90	pTDH3 NLS-VP16-EL222 tSSA1 (pIB0120)	pUPR mScarlet-I tENO1 (pIB0115)	pLight alpha-prepro-alpha_amylase-mNeonGreen-3xFLAG tTDH1 (pIB0677)	Methionine
Parental accessory	yIB44	yIB32	pTDH3 NLS-VP16-EL222 tSSA1 (pIB0120)		pLight mScarlet-I tENO1 (pIB0113)	Leucine, Methionine
Accessory	yIB337	yIB44	pTDH3 NLS-VP16-EL222 tSSA1 (pIB0120)	2x(pTDH3 mCerulean tTDH1) (pIB0267)	pLight mScarlet-I tENO1 (pIB0113)	Methionine
mNeon HAC1 knockout	yIB343	yIB169	pTDH3 NLS-VP16-EL222 tSSA1 (pIB0120)	pUPR mScarlet-I tENO1 (pIB0115)	pLight alpha-prepro-mNeonGreen-3xFLAG tTDH1 (pIB0401)	Methionine
scFv HAC1 knockout	yIB344	yIB171	pTDH3 NLS-VP16-EL222 tSSA1 (pIB0120)	pUPR mScarlet-I tENO1 (pIB0115)	pLight alpha-prepro-scFv-mNeonGreen-3xFLAG tTDH1 (pIB0401)	Methionine

Supplementary note 2. Use of an accessory strain

The accessory strain is co-cultured in all experiments (except the control experiments) at an initial ratio of 1:10 with respect to the strain of interest. It is used to guarantee a minimal flow of media through the reactors since its growth is not affected by secretory burden, to quantify the actual induction demand in the strain of interest, and to assess its growth rate.

2.1 – Quantifying effective induction levels

The accessory strain has been constructed to achieve unbiased measurements of the induction levels actually perceived by cells. Small day-to-day variations have been observed with our platform. This could originate from variations in LED intensities, or more likely, from small differences in the placement of the sampling or air supply lines within the vessel. The accessory strain expresses a red fluorescent protein, mScarlet-I, whose expression is controlled by the EL222 optogenetic system as for the gene of interest in the strain under study. Therefore, at steady state, the expression levels of mScarlet-I in the accessory strain inform on the production rate of the POI in the strain under study.

To obtain a uniform scale for induction levels across the entire study, the measured levels have been normalized by the maximal value we obtained for induction levels in the study. Therefore, normalized induction levels range from 0 to 1 in all experiments.

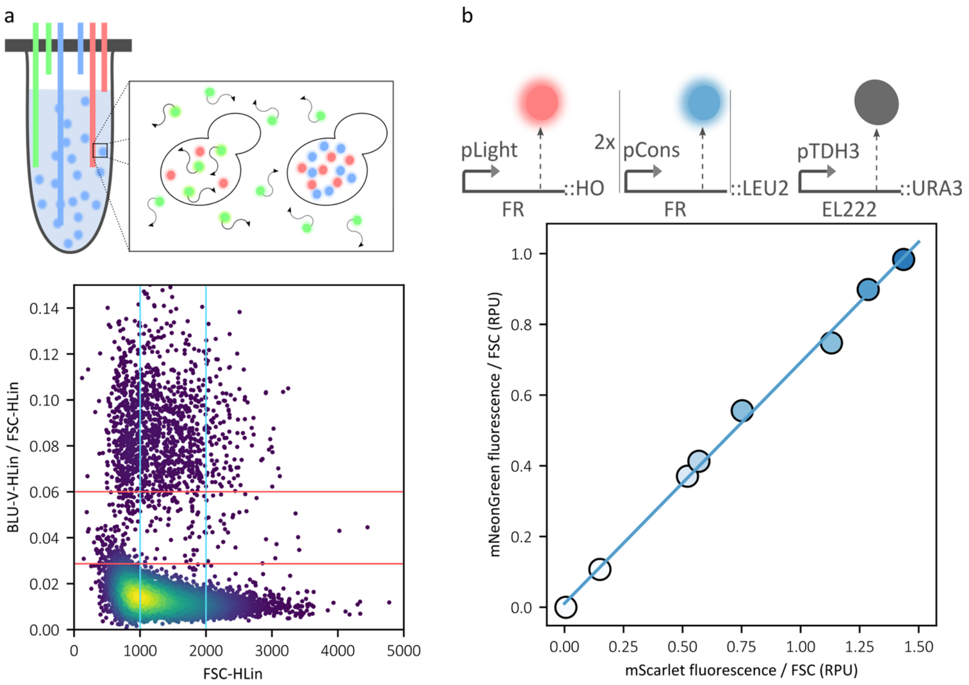

Fig. S2.1 Use of the accessory strain to assess induction levels. a The accessory strain is co-cultured together with the strain of interest. It can be differentiated because it contains 2 copies of mCerulean under the control of a constitutive promoter (pTDH3 from *S. cerevisiae*). The plot shows an example of gating of the accessory strain co-cultured with a strain expressing non-secreted mNeonGreen at maximal induction. The blue lines represent thresholds used to gate cells (1000 < FSC < 2000). Only events having values between 1000 and 2000 in FSC were kept to discard cell doublets and cell debris. The red lines represent the thresholds to differentiate each strain in the BLU-V channel after FSC normalization. Cells above the highest threshold (θ_{high}= 600) are considered as accessory strain cells. Cells below the lowest threshold (θ_{low}= 300) are considered as cells from the strain of interest. Events in between the two lines are not selected. **b** The accessory strain expresses a cytoplasmic red fluorescent protein, whose expression is controlled by the EL222 optogenetic system as for the gene of interest in the strain under study. The plot represents how the fluorescence detected for mScarlet-I in the accessory strain and for mNeonGreen in the non-secreted mNeonGreen-expressing strain correlate (R^2= 0.99). This confirms that the accessory cells can be used as a sensor of induction levels.

2.2 – Assessing growth rate dynamics

When working in turbidostat mode, the OD is kept constant and one can in principle infer the growth rate from the influx rate of the supply pumps. These estimates were not very precise, however. Here, we use changes in the relative abundance of the accessory strain with respect to the strain of interest. By starting with a known fraction of accessory strain cells in the population, it is possible to assess growth rate of the strain under study by following the temporal evolution of the ratio of the strains, assuming that the growth rate of the accessory strain remains constant. The slope of the log of the ratio of the two strain abundances provides the difference of the growth rates between strains. To obtain smooth estimates, we compute the slope using the 5 closest sampling time points, corresponding to an averaging window of approximately 4 hours.

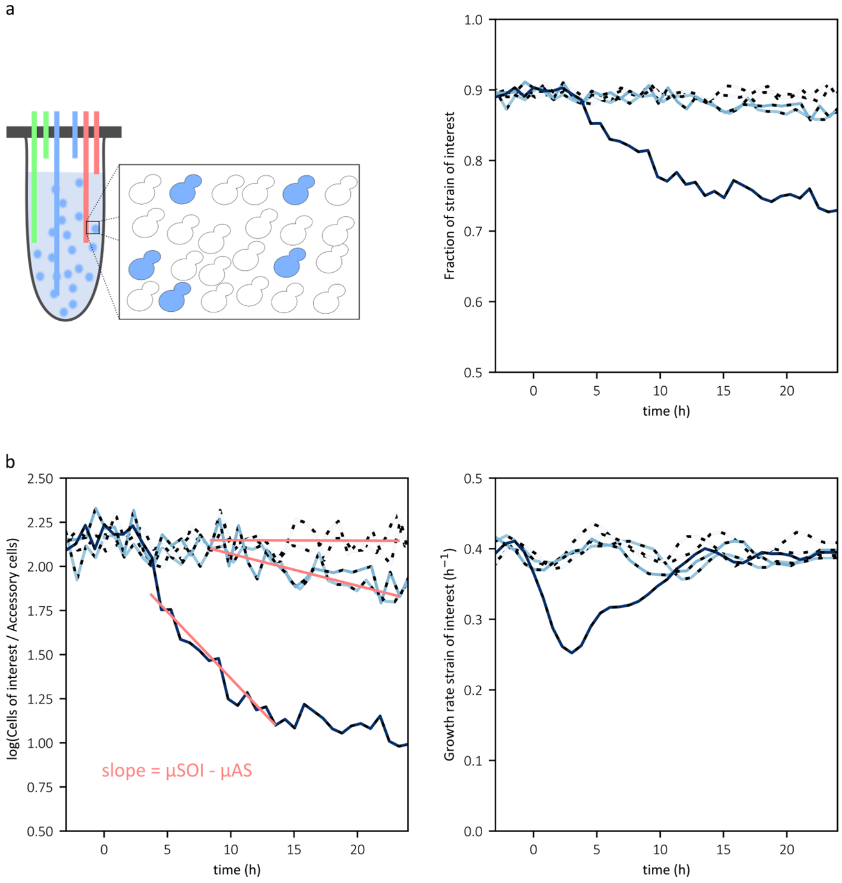

Fig. S2.2 Use of the accessory strain to assess the growth rate of the strain of interest. a A scheme of the co-culture of accessory strain and strain under study in the bioreactor is shown on the left. The accessory strain produces mCerulean that is used to differentiate it from the strain of interest and monitor the dynamic changes of its fraction. On the right, we represent the ratio of the amylase-secreting cells in the total population as a function of time and for different induction strengths. **b** Computation of the growth rate of the strain of interest using the temporal evolution of the ratio of the two strains. The selected data for this example corresponds to the amy-secreting strain, in ascendant order the induction levels are: 0, 0, 0.34, 0.37 and 1. µ: growth rate; SOI: strain of interest; AS: accessory strain

Supplementary note 3. Analysis of iPOI location in the cells

With our constructs that use the secretion signal of the α-factor of *S. cerevisiae*, the synthesized peptide is translocated to the ER after full translation. However, this process requires the protein to be unfolded, and therefore non-fluorescent. Consistently, we have observed by fluorescence microscopy that the fluorescence is spread across the whole cell in the non-secreted mNeonGreen cells, whereas the it is located exclusively in what should secretory compartments (ER, Golgi, vesicles, vacuole, etc.) in the secreted mNeonGreen cells.

Non-secreted mNeonGreen Secreted mNeonGreen

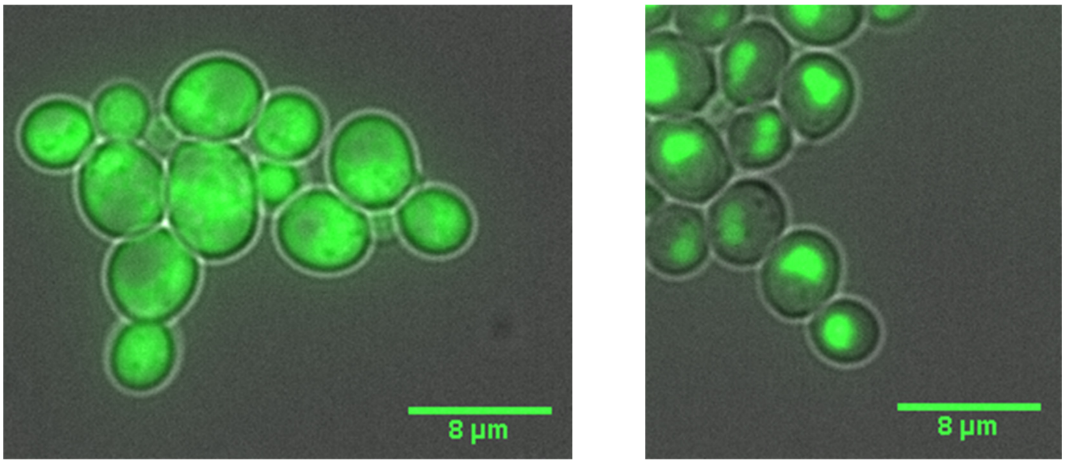

Fig. S3.1 Fluorescence microscopy images of yeast cells producing mNeonGreen, non-secreted and secreted. On the left, cells expressing non-secreted mNeonGreen and on the right, cells expressing the secreted protein. In both cases the expression was induced by constant light. Cells shown in the figure are representative of the cells in the field of view. Observations have been done twice with similar results.

Supplementary note 4. Quantification of secreted protein levels using magnetic immune beads

We provide details about the beads-based secretion measurements.

4.1 – Principle of secretion measurements

The goal is to develop a systematic approach to measure secretion levels of a wide range of proteins using a cytometer. To gather secreted proteins together so that they can be detected by the cytometer, we developed a method in which agarose microbeads bound to antibodies recognize and bind a common epitope in all secreted POIs, the FLAG tag. By incubating a sample of the culture with the immuno-beads, only the secreted proteins have access to bind the beads, whereas those inside the cells cannot interact with the antibodies. The procedure works as follows: (i) a sample of the culture is incubated with the magnetic beads coated with the anti-FLAG antibody, (ii) using a magnetic grid and washing steps, the cells are separated from the beads and the beads can be passed through a flow cytometer.

4.2 – Gating beads from residual cells

The distinction between cells and beads can be done by gating on the forward/side scatter pattern (FSC and SSC, respectively), since this property varies between cells and beads. The ratio SSC/FSC > 10 is used to distinguish beads from cells. We also gate to discard events having an SSC higher than the maximal SSC value (10^4) observed for cells (Figure S4.1).

For the optimization of the method, different types and concentrations of beads were tested for sufficient binding properties and for not clogging the flow cytometer capillary. To test the binding of the beads and the feasibility of the method, the first assays were performed with pure commercial GFP fused to the FLAG tag at C-terminal in different concentrations (Figure S4.1). The results show a linear relationship between GFP concentrations and fluorescence readings on the cytometer, with the minimum concentration in the linear range being 0.02 nM, and the maximum being 90nM.

Then, we tested supernatants of batch culture after 24 hours of induction and containing residual cells, in which the concentrations of mNeonGreen were unknown. The fluorescence signal from the beads was proportional to the dilution factors of the supernatant in pure water. Finally, we checked whether this methodology is sensitive enough to measure the secretion levels from samples directly taken from our continuous culture bioreactors. To do so, we took a volume of the cell culture directly from the reactors at different levels of light induction and after 24 hours of induction. Further information on beads measurements is provided in the thesis manuscript of Sebastian Sosa-Carrillo[7].

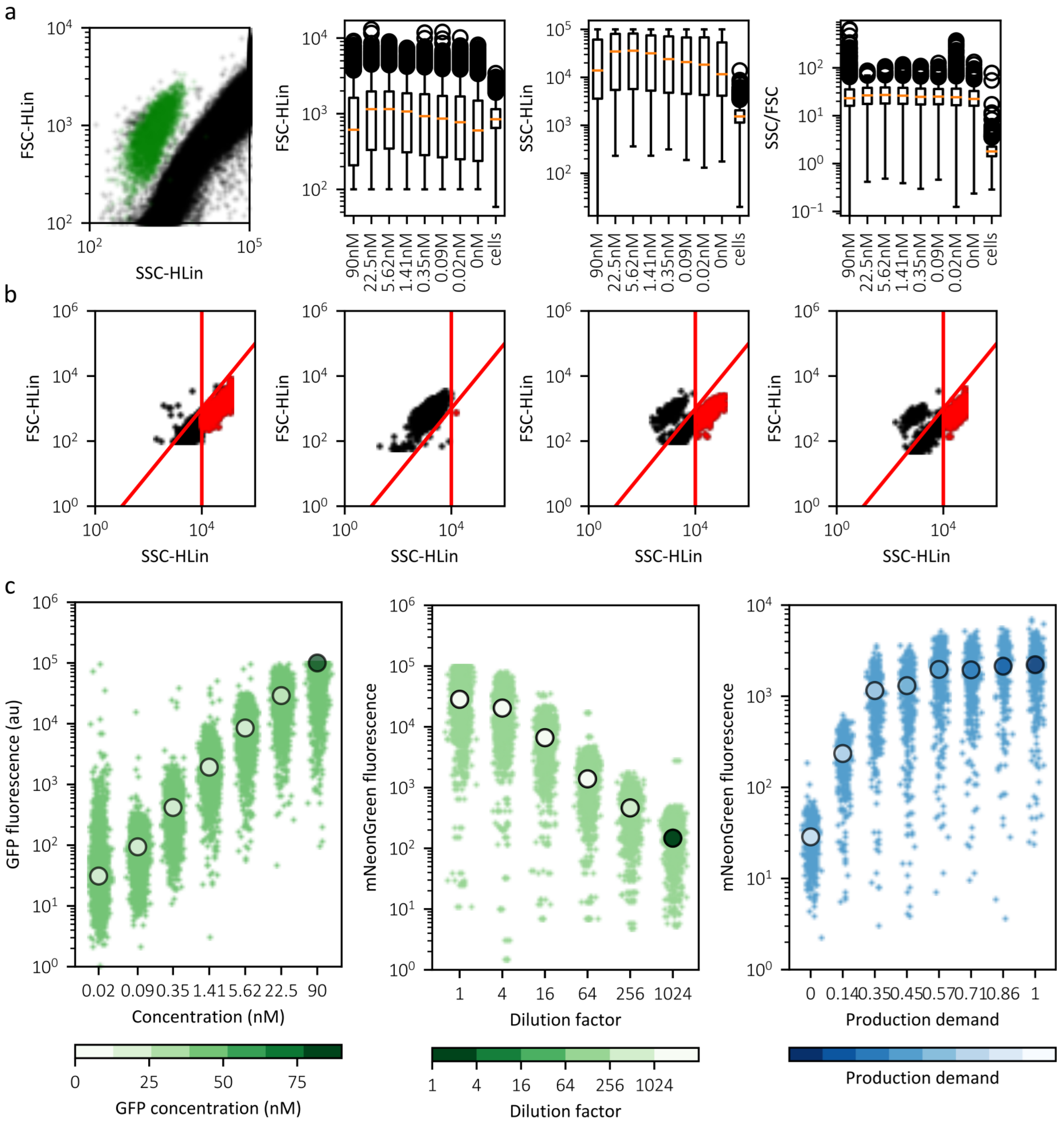

Fig. S4.1 Characterization of the secretion measurements performed by using immuno-magnetic beads. a On the left, we show the light scattering propertied of cells (green dots) and beads (black dots) from different experiments. On the right, the light scattering properties of beads with pure GFP and cells are represented in box plots. The data show that SSC and the ratio SSC/FSC are convenient for differentiation of both types of particles. In boxplots, the boxes represent the first to third quartile values of the data, with an orange line at the median. The whiskers cover the full range of the data. Outer points are data considered outliers. n= 5000 cytometry events for each box in boxplots. **b** Examples of the gating strategy from samples of different nature. From left to right, a sample containing only beads, a sample containing only cells, a sample from a batch culture containing both beads and cells, a sample from a continuous culture from our setup containing both beads and cells. The two samples from cell cultures are shown after washing most of the cells by the protocol explained in materials and methods of the main text. The red lines indicate the gating criteria, and the red dots are the gated events corresponding to beads. **c** Data obtained by the beads measurements protocol. On the left, the data obtained from assays with pure GFP-FLAG protein, where GFP-FLAG has been incubated at different concentrations. The middle plot represented the data obtained from a batch culture after 24 hours of maximal induction to secrete mNeonGreen-3xFLAG. The right plot corresponds to data obtained from a continuous culture after 24 hours of maximal induction to secrete mNeonGreen-3xFLAG. In all cases the small dots correspond to the beads after gating, and the large dots to the median of the population.

4.3 – Proteomics label-free quantification

We performed label-free quantitation of the POIs secreted in a continuous culture under full demand during 24 hours. Our goal was to define a coefficient to normalize the secretion levels measured with beads. These coefficients should account for possible differences in fluorescence levels coming from the structural context of the POI. Indeed, it is likely that proteins have different binding affinities for the beads.

The protein assay with the Pierce kit (660 nm for the determination) was performed on all 16 samples. 21 µg of protein was used per sample for the MS experiment. An equal amount of universal proteomic standard (UPS2, sigma) was added to all samples. Then, the samples were dried, resuspended, and denatured in 8M GuHCl, 5mM TCEP, 20mM ChloroAcetamide. Two successive protein digestions were performed. The first uses Endoprotease LysC at ratio E/S 1:50 (Promega) for 3 hours at 37°C. The samples were diluted five times in 50mM Tris pH8.0, and the second digestion was performed with trypsin at ratio E/S 1/50 (Promega) overnight at 37°C. Digestion was stopped adding 0.1% final of Formic acid (FA). The resulting peptides were desalted using a C18 cartridge.

LC-MS/MS analysis of digested peptides was performed on an Orbitrap Q Exactive Plus mass spectrometer (Thermo Fisher Scientific) coupled to an EASY-nLC 1200 (Thermo Fisher Scientific). A home-made column was used for peptide separation (C_{18}) 50 cm capillary column picotip silica emitter tip (75 µm diameter filled with 1.9 µm Reprosil-Pur Basic C_{18}-HD resin, (Dr. Maisch GmbH, Ammerbuch-Entringen, Germany)). It was equilibrated and peptides were loaded in solvent A (0.1% FA) at 900 bars. Peptides were separated at 250 nl/min. Peptides were eluted using a gradient of solvent B (ACN, 0.1% FA) from 3 to 7% in 8 min, 7 to 23% in 95 min, 23 to 45% in 45 min (total length of the chromatographic run was 170 min including high ACN level step and column regeneration). Mass spectra were acquired in data-dependent acquisition mode with the XCalibur 2.2 software (Thermo Fisher Scientific) with automatic switching between MS and MS/MS scans using a top 12 method. MS spectra were acquired at a resolution of 35000 (at *m/z* 400) with a target value of 3×10^6 ions. The scan range was limited from 300 to 1700 *m/z*. Peptide fragmentation was performed using higher-energy collision dissociation (HCD) with the energy set at 27 NCE. Intensity threshold for ions selection was set at 1×10^6 ions with charge exclusion of $z = 1$ and $z > 7$. The MS/MS spectra were acquired at a resolution of 17,500 (at *m/z* 400). Isolation window was set at 1.6 Th. Dynamic exclusion was employed within 45 s. Data were searched using MaxQuant (version 1.5.3.8) using the Andromeda search engine[8] against UPS2 proteins and mNeonGreen sequences. The following search parameters were applied: carbamidomethylation of cysteines was set as a fixed modification, oxidation of methionine and protein N-terminal acetylation were set as variable modifications. The mass tolerances in MS and MS/MS were set to 5 ppm and 20 ppm respectively. Maximum peptide charge was set to 7 and 5 amino acids were required as minimum peptide length. A false discovery rate of 1% was set up for both protein and peptide levels. The iBAQ intensity was used to estimate the protein abundance within a sample[9]. The match between runs features was allowed.

We obtained the iBAQ values, proportional to molar amounts of peptides in the sample, of the full mixture and also coverage of the corresponding POIs in each sample. Then, knowing the different abundance of the UPS2 peptides in the mixture, we could assess the abundance of our POIs (Figure S4.2, top). Normalization coefficients are defined as the ratio of the protein abundance estimated by proteomics over the fluorescence levels measured by beads. They are further rescaled so that the coefficient for the least abundant protein (scFv) is 1 (Figure S4.2, bottom and Table S4.1).
For a given protein, the secretion levels are then defined as the ratio of the measured beads fluorescence over the maximal beads fluorescence measured for this protein, scaled by its normalization coefficient.

Table S4.1 Coefficients used to normalize secretion levels.

Protein of interest	Name	pmoles/ml	Coeff
Secreted mNeonGreen	mNeon	47.5	11.6
Endo-1,4-beta-xylanase C	XylC	51.7	12.6
Endo-1,4-beta-xylanase 2	Xyl2	14.1	3.4
Paraoxonase 1	hPON1	5.7	1.4
Single chain variable fragment 4M5.3	scFv	4.1	1
α-amylase	amy	9.3	2.3

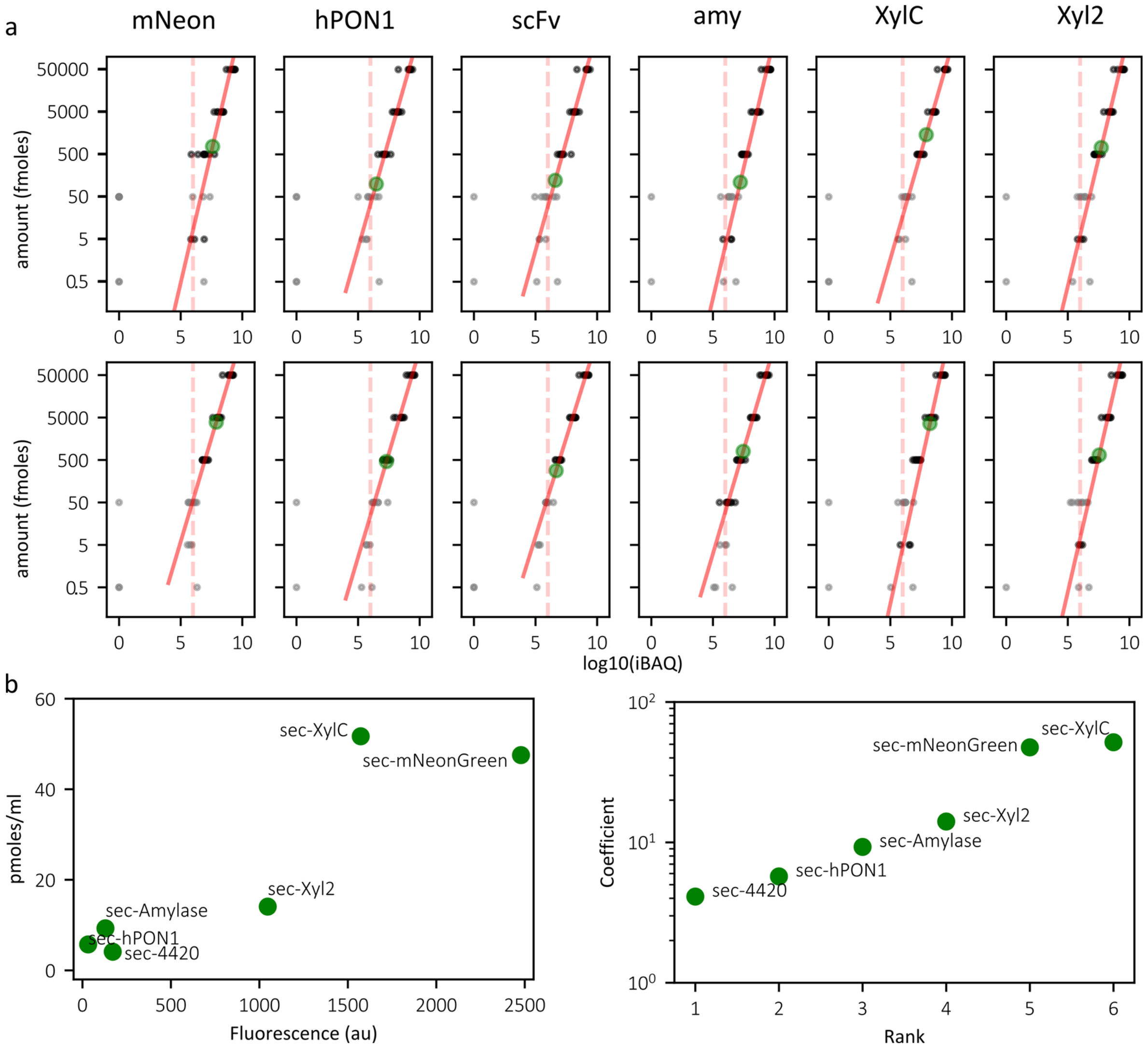

Fig. S4.2 Label-free quantification using proteomics results. a Comparison of label-free quantification of UPS2 proteins (black points) of known molar quantity (8 proteins per specified amount), with the POI (green points) in different experiments. To assess the molar quantity of the POI, we performed linear regression (red line) using the mean of the iBAQ values for the UPS2 proteins. Only means between 10^6 and 10^{10} were used for the regression, as indicated by the dotted red line. **b** On the left, the plot shows the relationship between the inferred molar quantity (average of two replicas) and the fluorescence obtained from the beads. On the right, we represent the coefficients for each of the POI that are used to normalize the fluorescence values from beads and obtain the secretion levels shown in this study.

Supplementary note 5. Complete time-course data for iPOI and UPR levels and analysis of the accumulator population

Here, we show complete time-course data for IPOI and UPR levels, and we provide details on the characterization of the accumulator cells performed in this study.

5.1 – Complete time-course data for iPOI and UPR levels

Because of space limitations, iPOI and stress levels are represented only for the first 15 hours in the main text (Figure 2). For completeness, we provide here the complete time-courses.

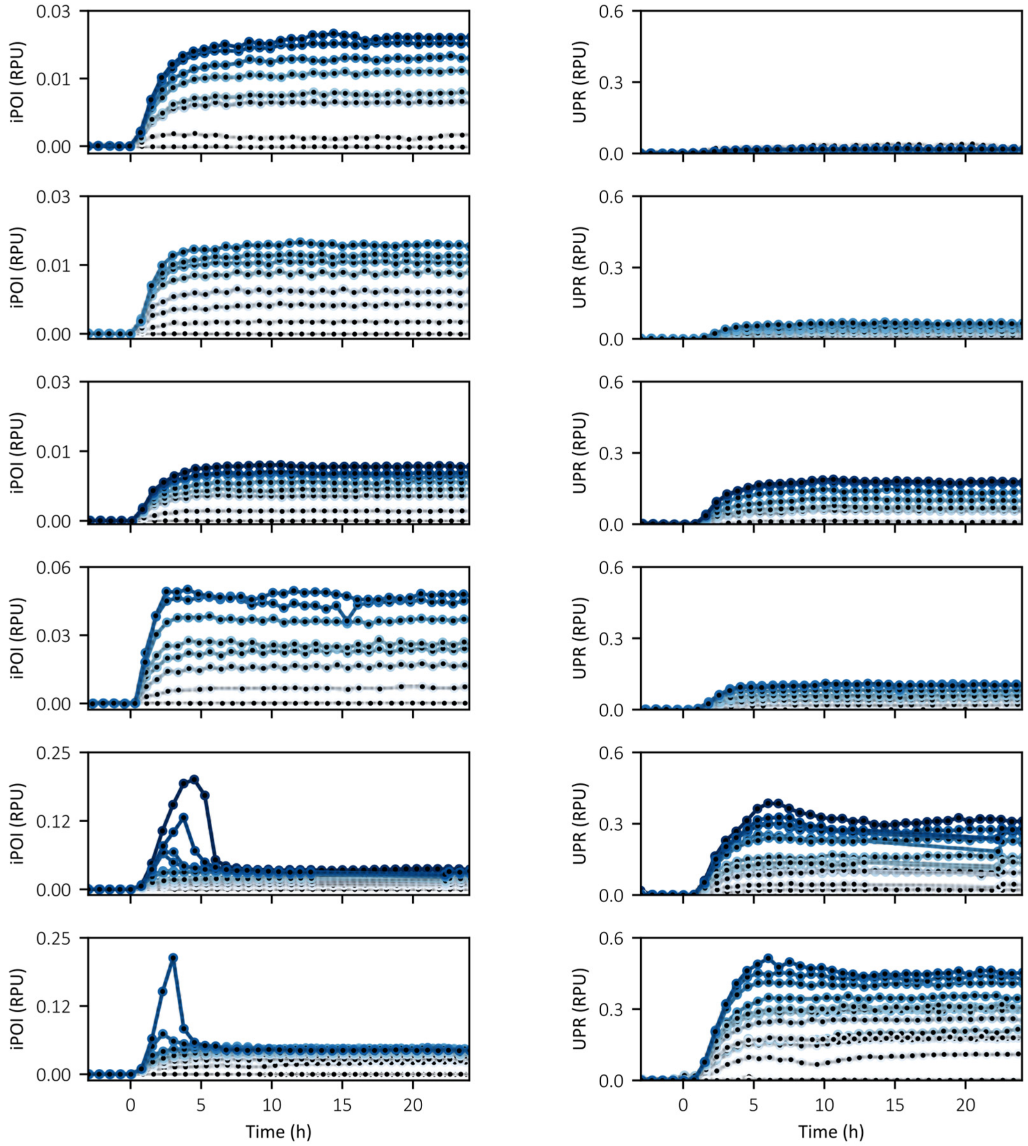

Fig. S5.1 Characterization of cellular trafficking capacities for six different POIs. Six strains secreting different POIs were subjected to different production demands. Their iPOI and stress levels were followed in time. Rows represent data for the different proteins (from top to bottom: mNeon, XylC, Xyl2, hPON1, scFv, amy). Columns represent, for different induction strengths, the temporal evolution of the median levels of iPOI distributions (left) and the temporal evolution of the median levels of UPR distributions (right). Induction levels are color-coded as represented in Figure 2 of the main text.

5.2 – Differentiating between accumulator and non-accumulator cells

To distinguish the two populations, we used a Gaussian mixture model provided in the Python package sklearn.mixture.GaussianMixture.

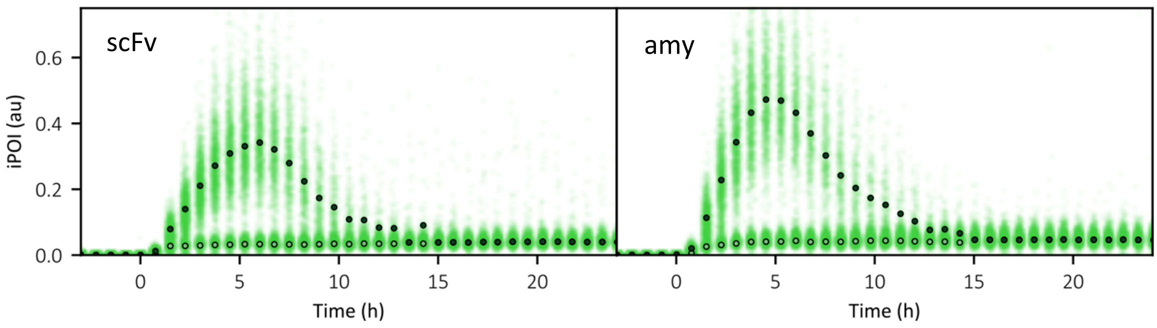

Fig. S5.2 Defining the accumulator and non-accumulator populations. The plot shows the two populations in two experiments. In each of them the temporal evolution of iPOI is shown over time. The median for accumulators at each time point is represented as a black dot, the median for non-accumulators as a white dot. The left plot corresponds to the experiment of scFv-secreting cells at maximal induction levels. The right plot corresponds to the experiment of amylase-secreting cells at maximal induction levels.

5.3 – Correlation of the maximal fractions of iPOI- and UPR-defined accumulators

The maximal fraction of accumulators defined by their iPOI levels is correlated with the maximal fraction of accumulators defined by their UPR levels. The main difference between these two maxima is the time at which they are reached.

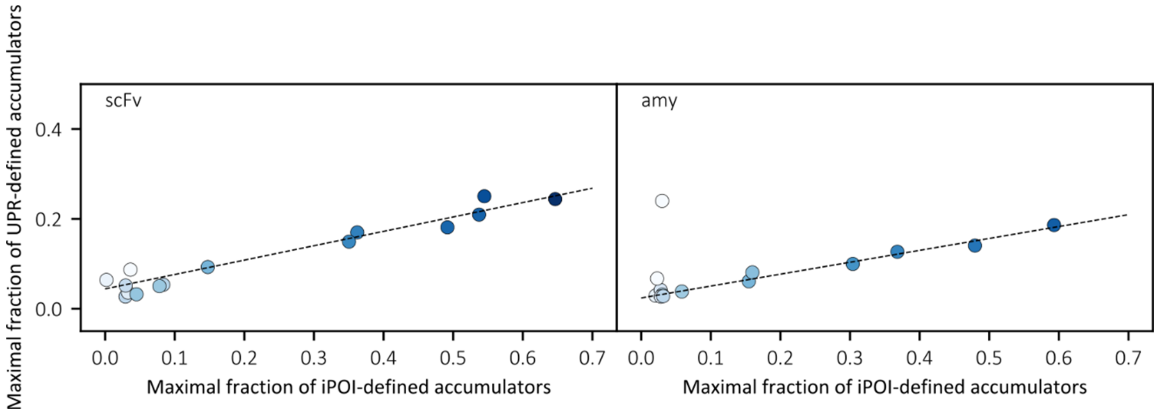

Fig. S5.3 Correlation between the maximal fractions of accumulators defined by their iPOI or UPR levels. In each plot the intensity of the blue color is proportional to the induction levels. The black line indicates the linear relation between the two variables.

Supplementary note 6. Analysis of cellular growth rates

We provide details on the characterization of the growth rate performed in this study and its relation with the presence of accumulator cells.

6.1 – Growth rates in characterization experiments

Here we show the growth rate for the characterization experiments shown in the main text for each of the proteins.

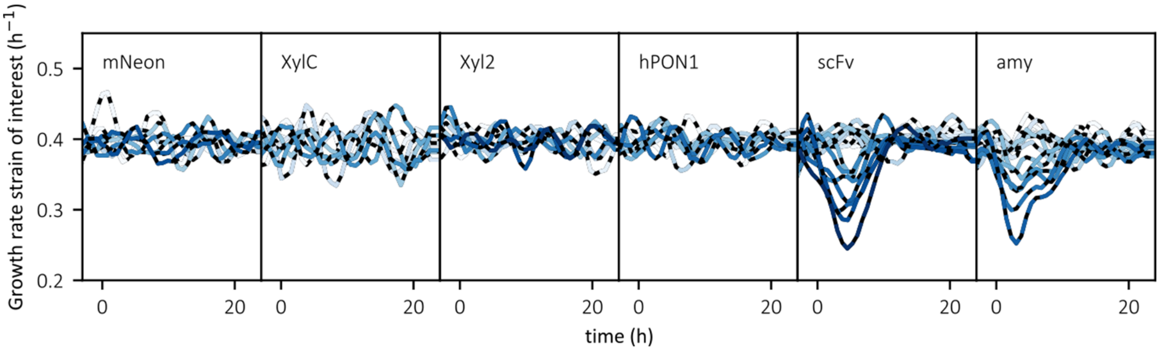

Fig. S6.1 Computed growth rate in characterization experiments. Each plot shows the growth rate corresponding to each strain of interest during the characterization experiments (from 3 hours prior to induction to 24 hours after induction) for all the different production demands. The intensity of the blue color corresponds to the induction levels.

6.2 – The fraction of accumulator cells is correlated in time with the overall growth rate

Here, we represent the relative decrease of the population growth rate at a given time ($\frac{\mu_{\text{ref}}-\mu(t)}{\mu_{\text{ref}}}$, with $\mu_{\text{ref}} = 0.4\ \text{h}^{-1}$) as a function of the fraction of accumulators in the entire cell population (strain of interest and accessory strain) at the same time. We observe that the growth rate of the population decays proportionally to the fraction of accumulators, indicating that accumulator cells have a strongly reduced growth rate.

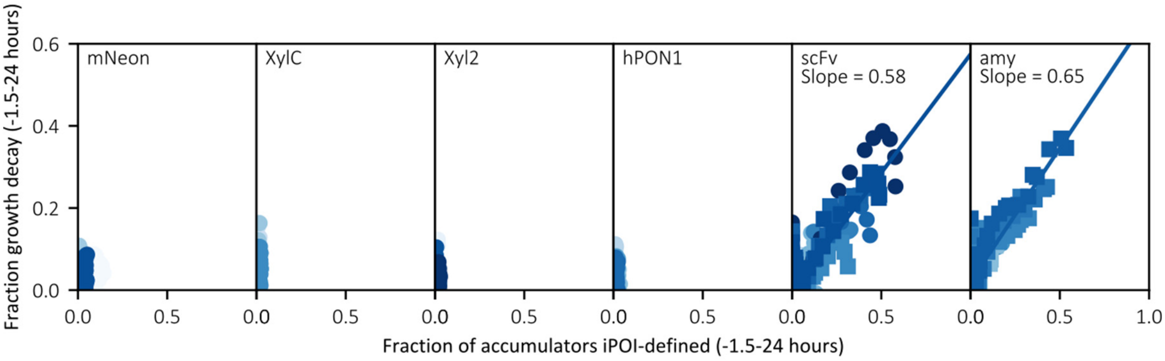

Fig. S6.2 Relation between the fraction of accumulators and the population growth decay. For each strain of interest, we show the relative decrease of the growth rate as a function of the fraction of the accumulator cells in the entire cell population during the characterization experiments (from 1.5 hours prior to induction to 24 hours after induction) for all the different production demands. The intensity of the blue color corresponds to the strength of the production demand. Circles and squares correspond to data obtained in two independent experiments. The slopes indicated for scFv- and amy-secreting cells have been computed by linear regression.

Interestingly, under the simple assumptions that non-accumulator and accessory cells grow normally at μ_{ref}, and that accumulator cells grow at a constant, decreased growth rate, μ_{acc}, it is easy to show

that the slopes represented in Fig 6.2 provide the relative decrease in growth rate of the accumulator cells, meaning that scFv and amy accumulators would grow 58% and 65% slower than non-burdened cells, respectively.

6.3 – Estimation of accumulator cells growth rates

From a dynamic perspective, when the demand is high for hard-to-secrete proteins, we observe that the proportion of accumulators first rapidly increases and second slowly reverts back to zero. This second phase is called an adaptation phase. The decrease of the proportion of accumulators can be explained by at least two phenomena. The internal level of the POI of the accumulators can decrease to levels that are typical to non-accumulator cells. This could result from the activation of stress adaptation responses such as protein degradation pathways. Alternatively, accumulators can be washed away from the bioreactors used in turbidostat mode since they grow slower than the non-accumulators and the accessory strain. One can see the first phenomenon as a cellular adaptation and the second phenomenon as a population adaptation.

To obtain an estimate of the relative importance of these two phenomena, we built a simple model that accounts only for population effects. Our key assumptions are that when the peak of accumulator fraction has been reached, (i) non-accumulators do not experience further secretion burn out (i.e. remain non-accumulators) and continue to grow normally, and (ii) accumulators do not adapt (i.e. remain accumulators) and grow significantly slower than non-accumulators. Taking into account the presence of cells from the accessory strain, we obtain the following model of differential equations:

$$\frac{dn_{acc}}{dt} = (\mu_{acc} - k_{dil}) \cdot n_{acc},$$

$$\frac{dn_{non-acc}}{dt} = (\mu_{non-acc} - k_{dil}) \cdot n_{non-acc},$$

$$\frac{dn_{acces}}{dt} = (\mu_{access} - k_{dil}) \cdot n_{access},$$

where n_{acc}, $n_{non-acc}$, and n_{access} are the numbers of accumulator, non-accumulator, and accessory cells, μ_{acc}, $\mu_{non-acc}$, and μ_{access} are the growth rates of accumulator, non-accumulator, and accessory cells, respectively, and k_{dil} is the dilution rate. We assume that $\mu_{non-acc} = \mu_{access} = \mu_{\mathrm{ref}}$. Given that the bioreactors are operated in turbidostat mode, we obtain for the dilution rate: $k_{dil} = \mu_{acc} \cdot \rho_{acc} + \mu_{\mathrm{ref}} \cdot (\rho_{non-acc} + \rho_{access})$, using ρ to denote cell proportions.

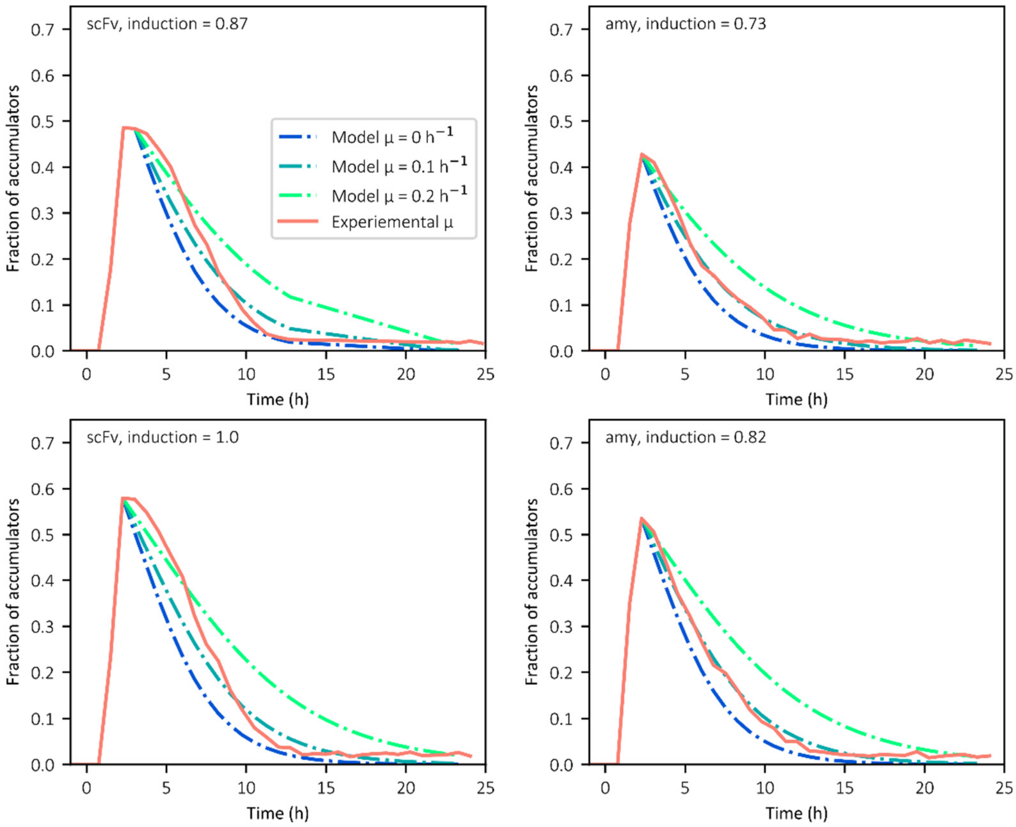

Fig. S6.3 Theoretical fraction of accumulator cells at different growth rates compared with experimental results. Predicted and observed fraction of accumulators in the populations of cells secreting scFv and amy (left and right columns, respectively), at the two highest levels of induction for each POI. The solid red line indicates the experimentally observed value in each of the experiments. The other plots represent the predicted fractions of accumulators for three different growth rates for accumulator cells, 0, 0.1 and 0.2 h^{-1}.

For the two POIs for which significant amounts of accumulators have been observed (scFv and amy), we computed model predictions for the evolution of the fraction of accumulators under different assumptions for the growth rate of accumulator cells (μ_{acc} being equal to $0\ \mathrm{h}^{-1}$, $0.1\ \mathrm{h}^{-1}$, or $0.2\ \mathrm{h}^{-1}$). These predictions are represented in Figure S6.3, together with the observed fraction of accumulators.

In the case of amy, the actual fraction of accumulators fits well the predicted values for a growth rate of 0.1 h^{-1}. Therefore, the observed behavior is perfectly compatible with a situation in which accumulators do not adapt, grow at a constant, low rate, and are all eventually washed away from the bioreactors. Yet, we cannot rule out other situations in which accumulators grow faster than $0.1\ \mathrm{h}^{-1}$ and adapt in part.

In the case of scFv, the decrease of the accumulator fraction is at some moments even faster than the decrease that is predicted in complete absence of growth. Therefore, it is highly likely that these cells adapt, at least in part, meaning that accumulator cells are not all washed away from the bioreactors.

Supplementary note 7. Characterization of *HAC1* knockout strains

We provide the iPOI and growth rate dynamics for the sets of experiments characterizing the Hac1 deficient cells secreting mNeon or scFv.

7.1 – mNeonGreen-secreting strain knockout

We provide the iPOI and growth rate dynamics for the experiment set corresponding to the Hac1 deficient cells secreting mNeon.

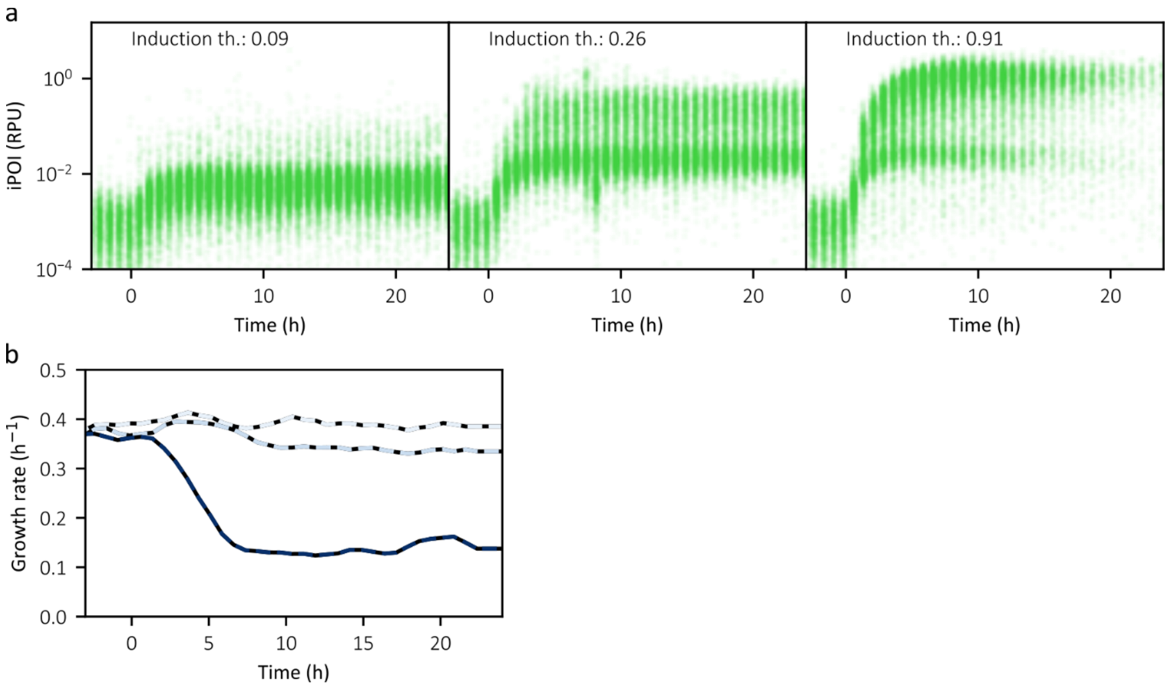

Fig. S7.1 iPOI and growth rate dynamics for mNeon Hac1-deficient population. a The plots show the resulting populations iPOI over time in each of the experiments for mNeon Hac1-deficient characterization. **b** In each plot is shown the growth rate corresponding to each of the experiments shown in a (from 3 hours prior to induction to 24 hours after induction) for all the different production demands. The intensity of the blue color corresponds to the production demand strength.

7.2 – scFv-secreting strain knockout

We provide the iPOI and growth rate dynamics for the experiment set corresponding to the Hac1 deficient cells secreting scFv.

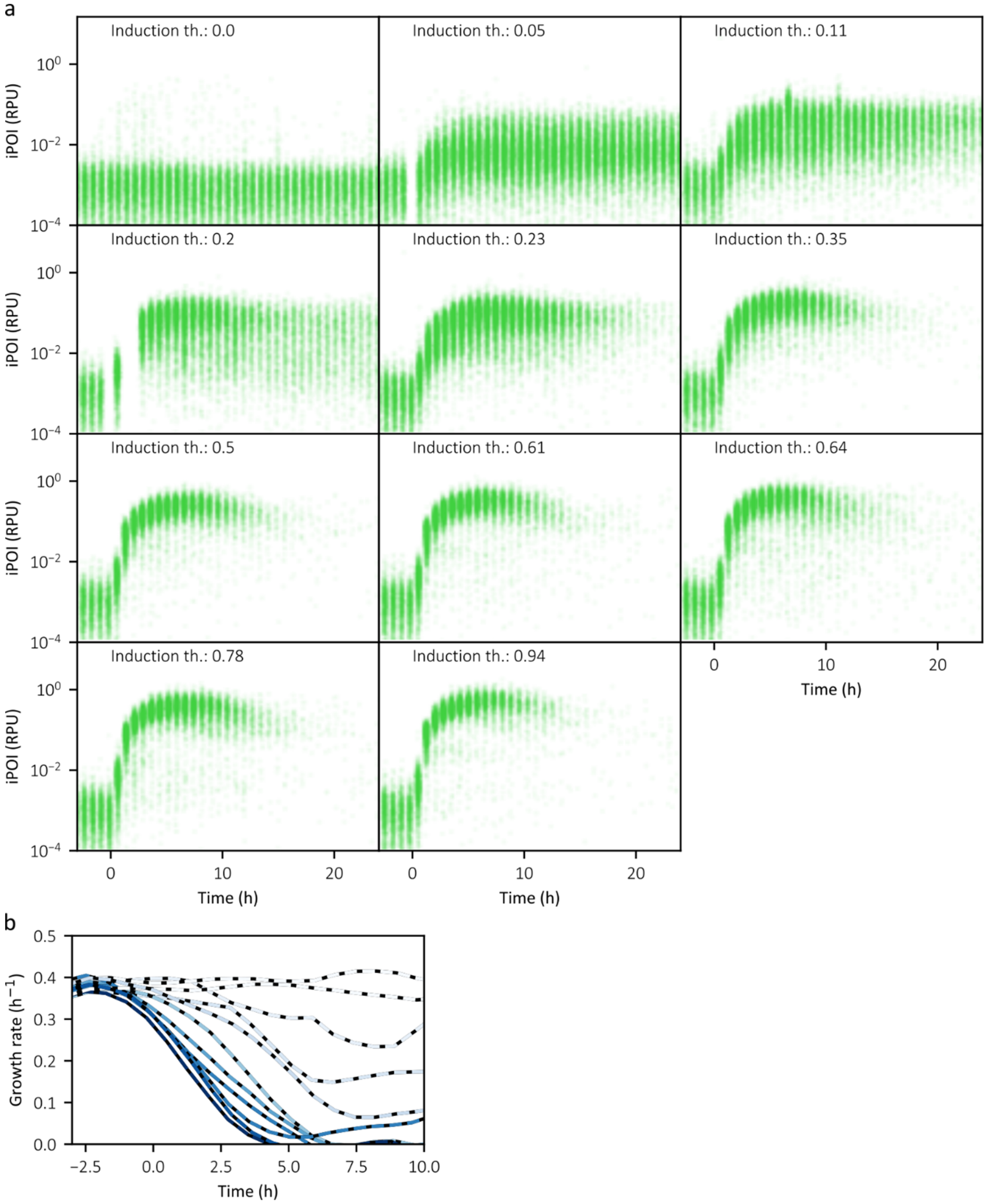

Fig. S7.2 iPOI and growth rate dynamics for scFv-secreting cells Hac1-deficient population. a The plots show the resulting populations iPOI over time in each of the experiments for scFv Hac1-defient characterization. **b** In each plot is shown the growth rate corresponding to each of the experiments shown in a (from 3 hours prior to induction to 24 hours after induction) for all the different production demands. The intensity of the blue color corresponds to the production demand strength. The data are shown for 10 hours of experiment because after that the strain of interest was diluted out of the reactors.

Supplementary note 8. ReacSight scripts for real-time control experiments

We provide a typical script used to define the control experiments using the ReacSight software. Details on the software and on the experimental platform can be found in Bertaux, Sosa, *et al.*, *Nat Commun*, 2022[10].

```
# extract last cytometer data
last_tp = state['current_tp']
data_last_tp = program.cells[program.cells['time_s'] == last_tp].copy()

# Process data befor event check
data_last_tp['ORG-G_norm'] = data_last_tp['ORG-G-HLin']

# Gate by size
data_last_tp_gated_size = data_last_tp[(data_last_tp['FSC-HLin'] > 1*10**3) & \
                                       (data_last_tp['FSC-HLin'] < 2*10**3)].copy()

# Normalize to size
data_last_tp_gated_size['BLU-V/FSC'] = data_last_tp_gated_size['BLU-V-HLin']/data_last_tp_gated_size['FSC-HLin']
data_last_tp_gated_size['ORG-G/FSC'] = data_last_tp_gated_size['ORG-G_norm']/data_last_tp_gated_size['FSC-HLin']

# Gate from accessory strain
data_last_tp_gated_from_sensor = data_last_tp_gated_size[data_last_tp_gated_size['BLU-V_FSC'] > 6*10**-2].copy()

# compute the UPR metric
UPR_level = data_last_tp_gated_from_sensor['ORG-G/FSC'].mean()
```

```
# changing light based on value
# should we increase the duty cycle ?
if UPR_level < pars['threshold']:
    state['current_dc'] += pars['DC']
    if state['current_dc'] > 1:
        state['current_dc'] = 1
# should we decrease the duty cycle ?
if UPR_level > pars['threshold']:
    state['current_dc'] -= pars['DC']
    if state['current_dc'] < 0:
        state['current_dc'] = 0

logging.info('T= {}| UPR feedback control reactor {}: UPR level = {}, new dc = {}'.format(time(),program.reactor_id,UPR_level,state['current_dc']))

# apply current dc
# start_LED_duty_cycle(intensity, period_s, fraction, n_cycles)
program.start_LED_duty_cycle(20, 45*60, state['current_dc'], 2000)

# update event state
state['last_change_tp'] = state['current_tp']
```

```
# Target value and DC step for reactor 1 (example)
targets_UPR_and_DC = {1:(0.17,0.05)}

# Call the function
for rid in [1]:
    thresh, DC = targets_UPR_and_DC[rid]
    event_UPR = Event(trigger=trigger_new_cyto_data,
                      action=action_UPR_ramp_feedback,
                      state={'last_change_tp':0, 'current_tp':0, 'current_dc':0.},
                      pars={'threshold':thresh, 'DC':DC})
    session.programs[rid].events.append(event_UPR)
```

Supplementary references

1. Shaner, N. C. *et al.* A bright monomeric green fluorescent protein derived from *Branchiostoma lanceolatum*. *Nat Methods* **10**, 407–409 (2013).
2. Do, T. T., Quyen, D. T., Nguyen, T. N. & Nguyen, V. T. Molecular characterization of a glycosyl hydrolase family 10 xylanase from *Aspergillus niger*. *Protein Expression and Purification* **92**, 196–202 (2013).
3. Torronen, A. *et al.* The two major xylanases from *Trichoderma reesei*: Characterization of both enzymes and genes. *Biotechnology* **10**, 1461–1465 (1992).
4. Ünver, Y., Kurbanoğlu, E. B. & Erdoğan, O. Expression, purification, and characterization of recombinant human paraoxonase 1 (rhPON1) in *Pichia pastoris*. *Turk J Biol* **39**, 649–655 (2015).
5. Boder, E. T., Midelfort, K. S. & Wittrup, K. D. Directed evolution of antibody fragments with monovalent femtomolar antigen-binding affinity. *Proc. Natl. Acad. Sci. U.S.A.* **97**, 10701–10705 (2000).
6. Liu, Z., Tyo, K. E. J., Martínez, J. L., Petranovic, D. & Nielsen, J. Different expression systems for production of recombinant proteins in *Saccharomyces cerevisiae*. *Biotechnol. Bioeng.* **109**, 1259–1268 (2012).
7. Sosa-Carrillo, S. Pipeline for the systematic characterization of heterologous protein secretory load to assess bioproduction efficiency. (Université Paris Cité, 2021). https://hal.science/tel-03612661
8. Tyanova, S., Temu, T. & Cox, J. The MaxQuant computational platform for mass spectrometry-based shotgun proteomics. *Nat Protoc* **11**, 2301–2319 (2016).
9. Schwanhäusser, B. *et al.* Global quantification of mammalian gene expression control. *Nature* **473**, 337–342 (2011).
10. Bertaux, F. *et al.* Enhancing bioreactor arrays for automated measurements and reactive control with ReacSight. *Nat Commun* **13**, 3363 (2022).
