# Supplementary material for: Maximizing protein production by keeping cells at optimal secretory stress levels using real-time control approaches
Source: Nat Commun. 2023 May 25;14:3028. doi: 10.1038/s41467-023-38807-9 (PMC10212943; doi:10.1038/s41467-023-38807-9)
Supplement: Supplementary file 2 — Reporting Summary [file 41467_2023_38807_MOESM2_ESM.pdf]

nature portfolio

Corresponding author(s): Gregory Batt

Last updated by author(s): Apr 25, 2023

Reporting Summary

Nature Portfolio wishes to improve the reproducibility of the work that we publish. This form provides structure for consistency and transparency in reporting. For further information on Nature Portfolio policies, see our Editorial Policies and the Editorial Policy Checklist.

Statistics

For all statistical analyses, confirm that the following items are present in the figure legend, table legend, main text, or Methods section.

n/a	Confirmed	
[ ]	[x]	The exact sample size (*n*) for each experimental group/condition, given as a discrete number and unit of measurement
[ ]	[x]	A statement on whether measurements were taken from distinct samples or whether the same sample was measured repeatedly
[x]	[ ]	The statistical test(s) used AND whether they are one- or two-sided<br>*Only common tests should be described solely by name; describe more complex techniques in the Methods section.*
[x]	[ ]	A description of all covariates tested
[x]	[ ]	A description of any assumptions or corrections, such as tests of normality and adjustment for multiple comparisons
[ ]	[x]	A full description of the statistical parameters including central tendency (e.g. means) or other basic estimates (e.g. regression coefficient) AND variation (e.g. standard deviation) or associated estimates of uncertainty (e.g. confidence intervals)
[x]	[ ]	For null hypothesis testing, the test statistic (e.g. *F*, *t*, *r*) with confidence intervals, effect sizes, degrees of freedom and *P* value noted<br>*Give P values as exact values whenever suitable.*
[x]	[ ]	For Bayesian analysis, information on the choice of priors and Markov chain Monte Carlo settings
[x]	[ ]	For hierarchical and complex designs, identification of the appropriate level for tests and full reporting of outcomes
[x]	[ ]	Estimates of effect sizes (e.g. Cohen's *d*, Pearson's *r*), indicating how they were calculated

Our web collection on statistics for biologists contains articles on many of the points above.

Software and code

Policy information about availability of computer code

Data collection	To grow cells, control gene expression, take samples and measure cell fluorescence by cytometry, we used an automated platform driven by the ReacSight software available at https://gitlab.inria.fr/InBio/Public/reacsight. ReacSight operations are described in a Jupyter notebook associated with each experiment. ReacSight notebooks are available online at doi:10.5281/zenodo.7418639. In addition to ReacSight (v1), we also used the OT-2 Python API (v2), and GuavaSoft (v3.3).
Data analysis	The Python code to process and analyze the raw data and generate figures for the manuscript can be found at https://gitlab.inria.fr/InBio/Public/yeastcybersecretion. It uses pandas (v1.0.2), numpy (v1.18.1), scipy (v1.4.1), sklearn (v0.22.1), cma (v3.0.3) and matplotlib (v3.1.3) packages.

For manuscripts utilizing custom algorithms or software that are central to the research but not yet described in published literature, software must be made available to editors and reviewers. We strongly encourage code deposition in a community repository (e.g. GitHub). See the Nature Portfolio guidelines for submitting code & software for further information.

Data

Policy information about availability of data

All manuscripts must include a data availability statement. This statement should provide the following information, where applicable:

- Accession codes, unique identifiers, or web links for publicly available datasets
- A description of any restrictions on data availability
- For clinical datasets or third party data, please ensure that the statement adheres to our policy

All the raw experimental data generated in this study have been deposited on Zenodo (doi.org/10.5281/zenodo.7418639) and Pride (https://www.ebi.ac.uk/pride/archive/projects/ PXD041650). Sequences of plasmids used to construct all yeast strains are available in the YeastCyberSecretion Git repository (https://gitlab.inria.fr/InBio/Public/yeastcybersecretion) in the GenBank format.

Human research participants

Policy information about studies involving human research participants and Sex and Gender in Research.

Reporting on sex and gender	Not applicable
Population characteristics	Not applicable
Recruitment	Not applicable
Ethics oversight	Not applicable

Note that full information on the approval of the study protocol must also be provided in the manuscript.

Field-specific reporting

Please select the one below that is the best fit for your research. If you are not sure, read the appropriate sections before making your selection.

☒ Life sciences ☐ Behavioural & social sciences ☐ Ecological, evolutionary & environmental sciences

For a reference copy of the document with all sections, see nature.com/documents/nr-reporting-summary-flat.pdf

Life sciences study design

All studies must disclose on these points even when the disclosure is negative.

Sample size	Thanks to automation, samples were taken and analyzed every 45 minutes. Samples were analyzed using cytometry (see below for details). 5000 events were recorded for each sample so that robust estimates of the mean have been obtained after gating.
Data exclusions	No data has been excluded for analysis.
Replication	Data is generated in an automated and relatively high-throughput manner. It is highly reproducible as documented in the ReacSight paper (https://doi.org/10.1038/s41467-022-31033-9). Each experiment tests 8 different conditions at a time.
Randomization	All experiments are fully automated so human bias in data production is absent.
Blinding	Blinding of the investigators during data collection has not been implemented since data collection is automated.

Reporting for specific materials, systems and methods

We require information from authors about some types of materials, experimental systems and methods used in many studies. Here, indicate whether each material, system or method listed is relevant to your study. If you are not sure if a list item applies to your research, read the appropriate section before selecting a response.

Materials & experimental systems

n/a	Involved in the study
☒	☐ Antibodies
☒	☐ Eukaryotic cell lines
☒	☐ Palaeontology and archaeology
☒	☐ Animals and other organisms
☒	☐ Clinical data
☒	☐ Dual use research of concern

Methods

n/a	Involved in the study
☒	☐ ChIP-seq
☐	☒ Flow cytometry
☒	☐ MRI-based neuroimaging

Flow Cytometry

Plots

Confirm that:

☒ The axis labels state the marker and fluorochrome used (e.g. CD4-FITC).

☒ The axis scales are clearly visible. Include numbers along axes only for bottom left plot of group (a 'group' is an analysis of identical markers).

☒ All plots are contour plots with outliers or pseudocolor plots.

☒ A numerical value for number of cells or percentage (with statistics) is provided.

Methodology

Sample preparation	Yeast cells were acquired directly from bioreactor cultures after dilution using DI water with the OT-2 robot.
Instrument	Guava EasyCyte 14 HT BGV
Software	Custom code was used for gating and is available in the git repository https://gitlab.inria.fr/InBio/Public/yeastcybersecretion.
Cell population abundance	We work with yeast cultures. No abundance issues were encountered.
Gating strategy	The gating strategy for cells is simple (two thresholds on FSC and on the blue fluorescence are used) and is documented in the Methods section of the main text and in Supplementary text 2. The gating strategy to distinguish beads from cells uses forward scatter and side scatter information and is documented in the Methods section of the main text and in Supplementary text 4.

☒ Tick this box to confirm that a figure exemplifying the gating strategy is provided in the Supplementary Information.
